# NURSE–FAMILY INTERACTION DURING ACUTE HOSPITALIZATION OF OLDER ADULTS

**DOI:** 10.1093/geroni/igae098.0773

**Published:** 2024-12-31

**Authors:** Orly Tonkikh, Elena Siegel, Yuuko Johnson, Everlyne Ogugu, Heather Young

**Affiliations:** University of California Davis, Davis, California, United States; University of California Davis, Davis, California, United States; University of California Davis, Davis, California, United States; University of California Davis, Davis, California, United States; UC Davis, Ashland, Oregon, United States

## Abstract

Family caregivers play a vital role for older adults during acute illness, yet there is limited research on the dynamics between nurses and families of older adults in acute care settings. This descriptive qualitative study explored perspectives on interactions during acute hospitalization in medical-surgical units among 24 nurses and caregivers. We conducted semi-structured interviews and used thematic analysis to identify key conditions and patterns of interaction. Nurses acknowledge the importance of family input to tailor and execute care plans to improve patient outcomes. Hospital structures (e.g., discharge planning guidelines) and nursing values and priorities (e.g., patient safety) influence the ways nurses interact with families. For example, nurses take a proactive approach in response to perceived safety and behavioral concerns and discharge planning. Without the structure of a formal assessment of caregiver needs and priorities, nurses rely on families to initiate interaction and glean insights about family context and preferences from anecdotal information. For the most part, nurses and families operate in parallel worlds with three main patterns of interaction: (1) caregiver proactivity leading to bi-directional interaction; (2) unidirectional interaction from nurses to caregivers; and (3) limited interaction. The study highlights lost opportunities for meaningful engagement to advance the older adult’s health. While nurses acknowledge the benefits of family engagement, few structures (e.g., training and standardized assessment) exist to facilitate family involvement in the current patient-centered healthcare system. This provides direction for further exploration of structures to support optimal nurse-family interaction, to enhance continuity and quality of acute care.

